# Comparing complications of urethral stricture across various ages: a retrospective analysis of findings from retrograde urethrogram and voiding cysto-urethrogram over 10 years

**DOI:** 10.1186/s12880-019-0384-7

**Published:** 2019-10-29

**Authors:** Ikenna I. Nnabugwu, Augustine C. Onuh, Solomon K. Anyimba, Samuel O. Mgbor

**Affiliations:** 1 Department of Surgery, College of Medicine, University of Nigeria Ituku-Ozalla, PMB 01129, Enugu, Nigeria; 2 Department of Surgery, University of Nigeria Teaching Hospital Ituku-Ozalla, Enugu, Nigeria; 3 Department of Radiology, University of Nigeria Teaching Hospital Ituku-Ozalla, Enugu, Nigeria; 4 Hansa Diagnostic Clinic, Plot 8A Ozubulu Street, Independence Layout, Enugu, Nigeria

**Keywords:** Urethral stricture, Complications, Late presentation, Age, Urethrogram

## Abstract

**Objective:**

To determine the rate and the distribution of the structural defects in the urinary bladder complicating urethral stricture in men, and to compare the complications observed in the younger patients to those observed in the older patients.

**Methods:**

Retrospectively, case files of patients diagnosed of urethral stricture using retrograde urethrogram (RUG) and voiding cysto-urethrogram (VCUG) from February 2009 to August 2018 were analyzed. Stricture characteristics were outlined. In addition, complicating structural defects in the lower urinary tract proximal to the stricture site were documented. The complicating defects identified in the patients were segregated according to age for any observable associations. Logistic regression analysis was used to define the nature of the association of patient age, stricture site, number and length, with distribution of complicating structural defects. Analyses were done using SPSS® version 20.

**Results:**

Within the 10-year review period, 257 of 421 suspected cases of urethral stricture were confirmed. Patients are between 1 and 104 years of age (mean: 50.1 ± 19.1 years; median: 51.0 years IQR 35.0–65.0). Bulbar (34.2%); short segment (62.6%); partial (73.9%) strictures are prevalent. Forty-seven (18.3%) of 257 patients presented with 1 or more complications. Bladder diverticulum (8.6%) and urethra-cutaneous fistula (6.6%) are prevalent complications. The distribution of complications does not vary with age, or with stricture characteristics.

**Conclusion:**

Presentation with complications is not uncommon. The distribution of these complications does not vary with age or with stricture characteristics.

## Introduction

Urethral stricture (US) is a known cause of infravesical obstruction. It develops as a consequence of healing with scaring of any significant inflammatory response of the urethra to any insult [1]. Such insult could be due to trauma, infection, allergy, caustics and so on [2]. Seen in almost all ages, US is predominantly a male disease condition especially among younger active men. Any part of the urethra from the prostatic urethra to the urethral meatus can be affected by this obstructing urethral disorder, but different parts predominantly manifest strictures from different aetiologic agents [3, 4]. Due to the peculiar mechanism of urethral injury in the posterior urethra (pelvic fracture urethral injury {PFUI}) and the absence of cavernous tissue in that segment of the urethra, strictures of posterior urethra are understood to differ from strictures of the anterior urethra [5]. Irrespective of the etiology, the mechanism of injury, the pathogenesis of the stricture and the anatomical site of the stricture, the resultant effect on urinary bladder emptying is the same: increased resistance to urine flow with recognizable consequences to the structure and function of the proximal urinary tract and the kidneys [6, 7].

These structural and functional abnormalities develop over a period determined by the evolution of the US. The resulting effect on the proximal urinary tract is dependent on the degree and the duration of increased resistance to urine flow due to the unyielding infravesical obstruction. In the urinary bladder and proximal urethra, such structural defects recognizable using a combination of retrograde urethrogram and voiding cysto-urethrogram include irregular inner bladder margin suggestive of thickened urinary bladder wall (detrusor hypertrophy) with implication similar to that of observing trabeculation and sacculation at cystoscopy [8], dilated proximal urethral segment and overdistended urinary bladder with significant postvoid residual urine volume [6]. Others include urinary bladder diverticulum of any size which could be solitary or multiple, vesico-ureteric reflux of any grade which could be unilateral or bilateral, and urethrocutaneous fistula of any course which could be solitary or multiple (watering-can perineum) [2, 9]. All of these are demonstrable with a combination of retrograde urethrogram and voiding cysto-urethrogram.

In addition to these structural defects, urinary calculi due to stasis of urine could be demonstrable [2]. Lower urinary tract calculi are by far commoner than upper urinary tract calculi in bladder outlet obstruction of various etiologies [8]. The presence of such lower urinary tract calculi is also revealed by retrograde urethrogram and voiding cysto-urethrogram.

As earlier indicated, these structural defects associated with bladder outlet obstruction of any etiology such as urethral stricture suggest prolonged increased infravesical resistance from urinary obstruction [10]. The rate of occurrence of these structural changes points towards late patient presentation in any medical care setting: settings observing many of these structural changes at presentation are dealing with many cases of late presentation. In addition, the type and distribution of the structural defects may vary from the younger to the older patient due to reported possible variations in bladder contractility with age [11, 12]. The aim of this study primarily is to determine the incidence rate of structural defects, beyond detrusor hypertrophy, in the lower urinary tract of patients who presented with radiologically confirmed urethral stricture. This information might serve as an index of late presentation with US in our setting. Secondarily, this study will give an insight into the existence of age-related differences in the type and distribution of structural defects complicating prolonged infravesical obstruction from urethral stricture.

## Methods

This is a retrospective analysis of case files of patients diagnosed of urethral stricture using retrograde urethrogram and voiding cysto-urethrogram from February 2009 to August 2018. From the case files, the site, length, number and completeness of the strictures were outlined. The structural changes in the lower urinary tract proximal to the stricture site detected through the contrast study were documented as well.

Subsequently, these detected lower urinary tract structural changes, proximal to the stricture site, were segregated according to age of patients for any significant associations. Finally, multivariate logistic regression analysis was used to define the nature of the associations between the predictor variables (age of patient, stricture site, stricture length and stricture completeness), and the dependent variable (types of complication). All analyses were done using SPSS® version 20 and the institution’s Bioethics Committee approved of the study.

## Results

There are 421 cases of suspected urethral stricture that underwent retrograde urethrogram (RUG) and voiding cysto-urethrogram (VCUG) within the 10 year review period. Of this lot, 257 are confirmed cases of urethral stricture which comes to an incidence rate of approximately 26 new cases of US per year from this center. The ages of the patients who own these case notes are from 1 to 104 years with a mean age of 50.1 ± 19.1 years and a median age of 51.0 (IQR 35.0–65.0). They are all males. The stricture characteristics are shown in Table 1.

**Table 1:** Anatomical characteristics of the strictures as detected through RUG + VCUG. Identified urethral strictures are more commonly solitary, short segment, incomplete and anterior in location

Variables	Frequency n (%)	
Stricture Site		
Glanular	6	2.3%
Penile	50	19.5%
Penobulbar	13	5.1%
Bulbar	88	34.2%
Bulbomembranous	20	7.8%
Posterior	77	30.0%
Panurethral	3	1.2%
Number of Stricture segments		
1	233	90.7%
> 1	24	9.3%
Length of Stricture		
Short (< 2 cm)	161	62.6%
Long (≥2 cm)	96	37.4%
Completeness of Stricture		
Partial	190	73.9%
Complete	67	26.1%

*RUG* retrograde urethrogram, *VCUG* voiding cysto-urethrogram

**Fig. 1:**
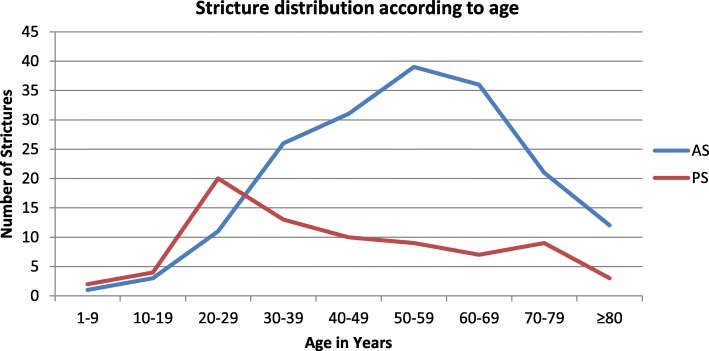
Frequency distribution of strictures in anterior (AS) and posterior (PS) urethra according to age of patients. The figure demonstrates that from the first decade of life, the number of patients presenting with complications of anterior urethral stricture increases up until the 6th decade of life. Beyond the 6th decade of life, the rates of presentation with complications decrease. On the other hand, presentation with complications from posterior urethral stricture peaks in the 3rd decade of life

The distribution of the strictures according to age of patients is shown in Fig. 1. Overall, the incidence of stricture peaks in the 6th decade of life. However, strictures in the posterior urethra are more prevalent in the younger patients (Pearson χ^2^ 30.27; df 8; *p* < 0.001).

Forty-seven (18.3%) of the 257 case files analyzed show evidence of complicating structural changes. The frequency distribution of these structural changes is shown in Table 2.

**Table 2:** Frequency distribution of detected complications of urethral stricture diagnosed with RUG + VCUG. There are 59 observed complications distributed among 47 of the 257 patients

Complication	Frequency n (%)	
Bladder Calculus	10	3.89%
Bilateral Vesico-ureteric Reflux	5	1.95%
Unilateral Right Vesico-ureteric Reflux	3	1.17%
Unilateral Left Vesico-ureteric Reflux	2	0.78%
Bladder Diverticulum	22	8.56%
Urethrocutaneous Fistula	17	6.61%

*RUG* retrograde urethrogram, *VCUG* voiding cysto-urethrogram

**Fig. 2:**
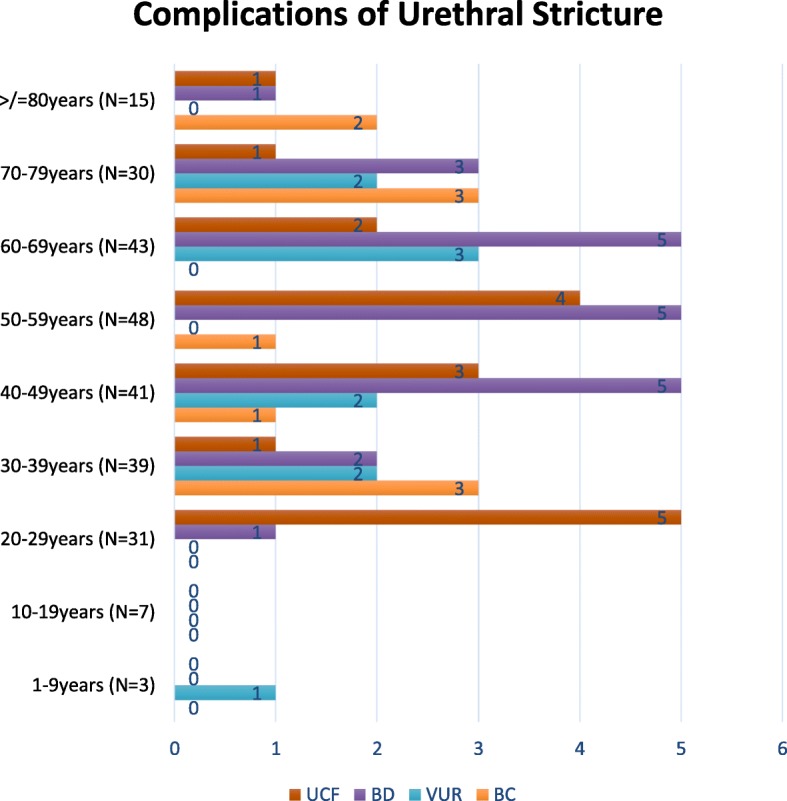
Frequency distribution of detected complications according to age groups. The figure demonstrates that there are no significant differences in the distributions of the observed complications across all age groups. Bladder diverticulum tend to be observed more frequently than other complications

Analysis of the detected complications according to age groups of patients shows that 18 (18.2%) of the 99 patients less than 45 years of age presented with structural changes while 29 (18.4%) of 158 patients 45 years and older also presented with detected structural changes (χ2 0.001; df 1; p 1.0). Further breakdown of detected structural changes according to age is shown in Fig. 2. There is no significant difference in the distribution of the complication across all age groups.

Further analysis with logistic regression shows that there are no indications of any significant predictions by age of patient (p 0.34), location of stricture (p 1.00), length of stricture (p 0.38) or completeness of stricture (p 0.88) of the presence or the distribution of complications.

## Discussion

The age distribution of patients in this study is similar to that from studies from other parts of Nigeria [13–15] and beyond [16, 17]. Though all age groups are involved, there is a predominance of men in the middle ages. In similarity to the reports from other studies [14, 15, 17], Table 1 shows that majority of the urethral strictures are located in the anterior urethra, especially the bulbar segment, and are short segment and incomplete strictures. In the absence of complicating structural changes due to delayed medical care, repair of strictures with such characteristics is expected to give good outcome [18].

Increased resistance to bladder evacuation due to significant infravesical obstruction results in structural changes in the part of the urinary tract proximal to the obstruction [2]. These structural changes defined with the aid of retrograde urethrogram and voiding cysto-urethrogram, and which may be indices of delayed presentation to appropriate medical care, can be seen across all ages. Irrespective of the age of patients, approximately 1 in every 5 patients (18.3%) from this study presented with one or more structural changes as complications of the existing urethral stricture. This reveals therefore, that patients present with avoidable complications of urethral stricture, with attendant possible negative effect on repair outcome.

Strictures in the posterior urethra are seen more commonly in the younger age groups, as is the finding from this study (Fig. 1), majorly because such strictures are usually due to high energy traumatic impact fracturing the bony pelvis, to which younger men tend to be more predisposed to due to involvement in activities with increased risks of such high energy traumatic impact [5, 19]. Correcting for age of patient, length, number and completeness of urethral stricture, there is no indication that the location of stricture along the urethra significantly predicts the occurrence of complicating structural changes. This observation is understandable recognizing that the common denominator to such complications is prolonged mechanical obstruction to urinary bladder evacuation.

Of the complicating proximal structural changes identified (Table 2) vesico-ureteric reflux appears to be very uncommon while bladder diverticulum and urethro-cutaneous fistula tend to be commoner, as can also be deduced from the work of Mungadi and Ntia [20]. It appears that the competent uretero-vesical junction does not yield with ease with persisting infravesical obstruction in the older patient. This corroborates earlier retrospective descriptive studies independently undertaken by Olajide et al. in Nigeria [14] and Fall et al. in Senegal [21] where no mention was made of any patient presenting with vesico-ureteric reflux among the many patients presenting with complications. On the contrary, reports on children presenting with vesico-ureteric reflux complicating infravesical obstruction from posterior urethral valve are not uncommon [22, 23].

Microscopically, some age-related changes have been reportedly observed in obstructed urinary bladder in animal studies [24, 25]. There is no indication from this study however that the resultant clinically obvious effect of such complicating structural changes varies significantly with age in urethral stricture patients (Fig. 2). The implication therefore, is that though there may be histologic and physiologic differences in the response of the urinary bladder to prolonged infravesical obstruction with respect to age in animal studies [25], clinically obvious proximal structural changes due to urethral stricture, a cause of infravesical obstruction, may not vary with age of patients in humans. Whether this finding is peculiar to urethral stricture or applies to other known causes of mechanical infravesical obstruction needs further studies to be established. So far, this study has revealed in real life terms, with no violation of ethical standards, the possible effect of persisting mechanical infravesical obstruction from urethral stricture in human males.

In conclusion therefore, available information from this retrospection suggests that across all ages, the development of clinically obvious structural changes in the lower urinary tract proximal to site of urethral stricture does not depend on the site of stricture, the length of stricture or the number of stricture segments. This is despite the reported observation that there are significant age-related microscopic differences in urinary bladder reaction to infravesical obstruction. Noteworthy also is the finding that vesico-ureteric reflux is uncommon as a complication of urethral stricture across all ages, but especially in the older patients.

### Limitation of this study

Data on duration of lower urinary tract symptoms attributable to infravesical obstruction from urethral stricture was either unavailable or unreliable for majority of the case notes, and was not included in the study.

## Data Availability

Data supporting this manuscript is available at https://data.mendeley.com/drafts/. doi: 10.17632/yxdf52vztg.1
